# Personalized electro-acupuncture versus auricular-acupuncture comparative effectiveness (PEACE): A protocol of a randomized controlled trial for chronic musculoskeletal pain in cancer survivors

**DOI:** 10.1097/MD.0000000000020085

**Published:** 2020-05-22

**Authors:** Kevin T. Liou, Ray Baser, Sally A.D. Romero, Jamie Green, Q. Susan Li, Irene Orlow, Katherine S. Panageas, Jun J. Mao

**Affiliations:** a Integrative Medicine Service, Department of Medicine; b Department of Epidemiology and Biostatistics, Memorial Sloan Kettering Cancer Center, New York, NY; c Department of Family Medicine and Public Health, UC San Diego School of Medicine, San Diego, CA.

**Keywords:** cancer, acupuncture, chronic pain, comparative effectiveness, pain management, personalized medicine

## Abstract

**Introduction:**

Chronic pain is a leading cause of disability and remains under-treated in nearly half of patients with cancer. The opioid crisis has highlighted an urgent public health need for effective nonpharmacological pain management. Electroacupuncture (EA) and Battlefield Acupuncture (BFA) represent nonpharmacological modalities used in clinical practice to manage pain; however, their effectiveness has not been rigorously evaluated in oncology settings.

**Methods:**

We describe the design of a 3-arm, parallel, single-center, multisite randomized controlled trial that investigates EA and BFA versus usual-care wait-list control (WLC) for chronic musculoskeletal pain among 360 patients with diverse cancer types across various stages. The primary aim is to compare effects of EA and BFA versus WLC on pain, physical function, and co-morbid symptoms. The secondary aim is to examine the interaction between patient outcome expectancy and acupuncture modality (EA vs BFA) on pain reduction. The tertiary aim is to evaluate the association between genetic polymorphisms and responses to acupuncture. Patients will be randomized in a 2:2:1 ratio to EA:BFA:WLC. Acupuncture groups will receive weekly treatments over 10 weeks. WLC will receive usual care over the same evaluation period as the acupuncture groups. The primary endpoint will be the change in average pain intensity score from baseline to week 12. We will collect validated patient-reported outcomes and blood/saliva samples at multiple timepoints over 24 weeks.

**Discussion:**

Our findings will advance nonpharmacological pain management in oncology and inform personalized treatment approaches that integrate individuals’ expectations and genetic biomarkers to deliver “precision” acupuncture to cancer patients with chronic pain.

**Trial Registration:**

ClinicalTrials.gov Identifier: NCT02979574

## 1 Introduction

Chronic pain affects approximately 100 million Americans and is the leading cause of disability in the United States.^[1]^ Compared to the general population, patients with cancer experience significantly greater pain burden,^[2]^ yet approximately 1 in 2 remain under-treated for pain,^[3,4]^ contributing to poor quality of life, impaired physical functioning, and worse cancer-related outcomes and overall survival.^[5]^ The national opioid crisis has created new challenges to pain management in the cancer population^[6]^ and highlighted the urgent public health need for effective nonpharmacological therapies.^[7]^

Derived from Traditional Chinese Medicine, acupuncture is a therapeutic modality that involves insertion of thin, sterile, single-use, metallic needles into the body surface.^[8]^ It is considered safe with few side effects.^[9,10]^ Acupuncture has been shown to modulate ascending and descending pain pathways in animal models,^[11–13]^ and human functional neuroimaging studies have elucidated the effects of acupuncture on key brain areas involved in pain processing.^[14]^ A recent meta-analysis demonstrated a moderate level of evidence of acupuncture for cancer-related pain; however, there was substantial heterogeneity among the studies and the types of acupuncture techniques that were evaluated.^[15]^

Electroacupuncture (EA) represents a specific acupuncture modality that involves electrical stimulation of needles, and its growing use in pain management is supported by scientific research demonstrating differential modulation of endogenous opioids by electrical stimulation of varying frequencies.^[16]^ Several trials have evaluated EA for chronic nonmalignant pain,^[17,18]^ but evidence for this particular modality remains limited in cancer populations.^[19]^ Battlefield Acupuncture (BFA), an auricular form of acupuncture developed by Colonel (Ret) Richard C. Niemtzow, MD,^[20]^ has received growing interest based on preliminary reports of its effectiveness for pain.^[21–23]^ Due to its standardized protocol and relative ease of administration, BFA is undergoing nationwide implementation within the Veterans Healthcare Administration (VA)^[24]^; however, paucity of rigorous effectiveness data remains a major barrier to successful implementation.^[25]^

We describe a randomized controlled trial that addresses these gaps in the evidence base of EA and BFA and seeks to evaluate their effectiveness compared to usual care for chronic pain in oncology settings. In light of the growing emphasis on personalized pain management,^[26]^ our trial will also seek to understand patient-level characteristics, both psychological (ie, expectancy) and biological (ie, genetic biomarkers), that can be leveraged to predict individual response to acupuncture. Our primary aim is to compare the effects of EA and BFA versus usual-care waitlist control (WLC) on patient-reported pain, physical function, and co-morbid symptoms in cancer patients with chronic musculoskeletal pain (Aim 1). Our secondary aim is to examine the interaction between patient outcome expectancy and acupuncture modality (EA vs BFA) on pain reduction (Aim 2). Our tertiary aim is to evaluate the association between genetic polymorphisms and responses to acupuncture (Aim 3). The findings will inform the evidence-based, personalized delivery of acupuncture to improve pain management for patients with cancer in the era of the opioid epidemic.

## 2 Methods

### 2.1 Study design

**Figure 1:**
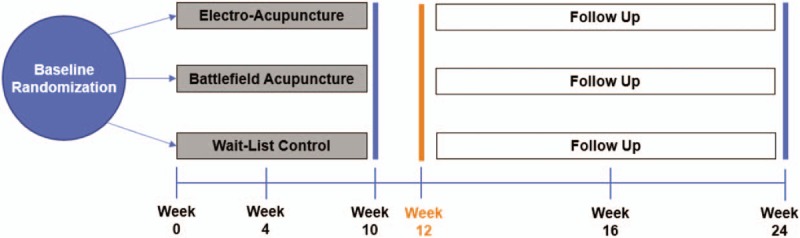
Study Schema.

This study is a 3-arm, parallel, single-center, multisite randomized controlled trial (RCT) to evaluate the comparative effects of EA and BFA versus WLC on pain and co-morbid symptoms in a heterogenous sample of 360 patients with cancer who are experiencing chronic musculoskeletal pain (Fig. 1). Eligible patients will be randomized in a 2:2:1 ratio to EA, BFA, and WLC groups. The interventions (EA and BFA) will be delivered once a week over 10 weeks for a total of 10 treatments. The WLC group will receive standard pain management (eg, analgesics, physical therapy, injections) over the same evaluation period as the acupuncture groups. The primary endpoint will be the change in average pain intensity score (as assessed by the Brief Pain Inventory) from baseline to Week 12. We will also collect validated patient-reported measures of pain interference, physical function, co-morbid symptoms (fatigue, psychological distress, post-traumatic stress disorder, and sleep disturbances), outcome expectancy, quality of life, and healthcare expenditures, as well as serum blood or salivary samples for genotyping, at multiple timepoints over 24 weeks (Table 1).

**Table 1:**
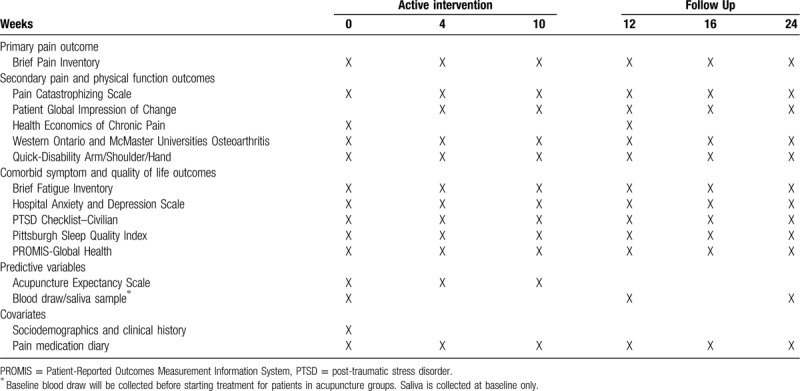
Data collection schedule.

To enhance the rigor and transparency of our study, we will adhere to the guidelines from Consolidated Standards of Reporting Trials (CONSORT)^[27]^ and Standards for Reporting Interventions in Clinical Trials of Acupuncture (STRICTA).^[28]^ The trial is funded by the U.S. Department of Defense (W81XWH-15-1-0245) and is registered at ClinicalTrials.gov (Identifier: NCT02979574). All study procedures are approved by the institutional review board at Memorial Sloan Kettering Cancer Center (MSK).

### 2.2 Participants

Study participants will be recruited from MSK, a National Cancer Institute-designated comprehensive cancer center with a main campus located in Manhattan and multiple other regional sites throughout New York (Westchester County, Long Island) and New Jersey (Bergen County, Monmouth, Basking Ridge). Recruitment will take place at a total of 6 MSK sites across diverse regions in New York and New Jersey. We will primarily use a population-based recruitment strategy by mailing letters to potentially eligible participants identified through MSK's patient registry. We will also engage oncology stakeholders to publicize the study and facilitate referrals. The target accrual goal is 360 participants. Enrollment began in March 2017 and study participant assessments are scheduled to be completed in May 2020.

### 2.3 Inclusion and exclusion criteria

English-speaking adult patients with a primary diagnosis of any cancer type will be eligible for the study. Patients with history of metastatic cancer are eligible if they have no current evidence of disease. Active treatment with surgery, chemotherapy, and/or radiotherapy must have been completed at least 1 month before study initiation; however, patients may continue to receive hormonal treatment or maintenance targeted therapies during the study.

To be eligible, patients must also report chronic musculoskeletal pain as the primary source of pain, defined as regional (eg, joints, extremities, back) or generalized (eg, fibromyalgia, diffuse body pain). This pain must be present for at least 3 months and for at least 15 days in the preceding 30 days. Their worst pain intensity in the preceding week must be rated as ≥4 on a 0 to 10 numerical rating scale. Nonmusculoskeletal pain syndromes (eg, headache, chest pain, visceral abdominal pain) may be present as co-morbid conditions if the patient reports musculoskeletal pain as the primary source of pain.

Patients will be excluded if they have inflammatory arthritis requiring disease-modifying drugs; phantom limb pain; a pending pain-related Veteran Administration, social security, or worker's compensation disability claim by self-report; or an implanted electronically charged medical device.

### 2.4 Procedure

All potential participants will undergo an initial screening with a research coordinator in person or over the phone. During this initial screening, the research coordinator will communicate study goals and procedures and screen participants for eligibility. A study clinician will then review screened patients to confirm that all inclusion/exclusion criteria are met. Once eligibility is confirmed, participants will complete informed consent and undergo randomization.

Participants will complete assessments at weeks 0, 4, 10, 12, 16, and 24. These assessments will be completed electronically online using REDCap electronic data capture tools.^[29]^ To encourage adherence to the study procedures, all participants will be compensated with a $30 gift card at weeks 0, 4, 10, 12, 16, and 24 for a total of $180. Additionally, participants will receive reminders to complete study assessments.

### 2.5 Randomization

We will randomize 360 participants using MSK's Clinical Research Database, a secure system that provides full allocation concealment and ensures that treatment group assignments cannot be guessed before or modified after a patient is enrolled in the trial. Group assignment (2:2:1 for EA:BFA:WLC) will be performed using permuted block randomization, stratified by accrual site (main campus vs regional sites) and baseline opioid use (yes vs no). The study statisticians will be blinded to treatment assignments.

### 2.6 Primary outcome

The Brief Pain Inventory (BPI) is one of the most widely used instruments to quantify pain intensity and interference. It is a reliable, valid measure with Cronbach α ranging from 0.77 to 0.91 and has been shown to be responsive to interventions.^[30]^ The BPI includes 4 questions related to the pain intensity with response choices ranging from 0 (“no pain”) to 10 (“pain as bad as you can imagine”). The change in average pain intensity score from baseline to week 12 will be the primary endpoint for this study. Patients who report a ≥30% reduction in their pain intensity will be classified as treatment responders who demonstrated a clinically meaningful improvement.^[31]^ In addition to the pain intensity items, the BPI also contains 7 pain interference questions with response choices ranging from 0 (“does not interfere”) to 10 (“completely interferes”); the average of these interference scores will be a secondary outcome.

### 2.7 Secondary pain and physical function outcomes

The Pain Catastrophizing Scale (PCS) is a validated, reliable 13-item scale (Cronbach α = 0.93) that assesses the negative cognitive-emotional responses to pain.^[32]^ The 13 items describe different thoughts and feelings that may be associated with pain symptoms. The PCS yields a total score and 3 subscales scores (ie, rumination, magnification, and helplessness).

The Patients’ Global Impression of Change is a 1-item survey that will be used to define a clinically important change from the patient's perspective.^[33]^ Patients will be asked, “How would you describe your pain since the first clinical visit?” with the following response choices: very much worse, much worse, a little worse, the same, a little improved, much improved, or very much improved. Patients reporting “much improved” and “very much improved” will be classified as treatment responders.

The health economics of chronic pain will be measured by assessing the monetary value of patients’ resource consumption during the 12-week period after randomization. Information on resource use will be collected by medical care cost questions^[34,35]^ and by calculating health insurance direct costs (eg, physician office visits, hospital stays, prescription drugs, acupuncture sessions) and indirect costs (eg, patients’ work incapacity). To evaluate the cost-effectiveness of acupuncture, we will collect quality-of-life data using the Patient-Reported Outcomes Measurement Information System-Global Health (PROMIS-Global Health) and convert these into Euro-QoL (EQ-5D) index scores.^[36]^ For the purposes of this study, the cost of each acupuncture session will be valued at $100 for a total of $1000 over 10 treatments.

The Western Ontario and McMaster Universities Osteoarthritis (WOMAC)–Physical Function subscale will be used to assess functional limitations of the lower extremity. This WOMAC subscale has been shown to be reliable (Cronbach α ranging from 0.92 to 0.97) and responsive to therapeutic interventions.^[37]^ The physical function subscale consists of 17 questions with excellent face, content, and construct validity. For each question, patients choose the degree of functional difficulty ranging from 0 (“none”) to 4 (“extreme”). These scores are summed to yield of total score 0 to 96 with higher scores indicating greater degrees of functional difficulties.

The Quick-Disability Arm/Shoulder/Hand (quick-DASH) is an 11-item instrument that measures disability related to arm, shoulder, and hand symptoms.^[38]^ Scores range from 0 (no disability) to 100 (most severe disability). We validated this instrument in patients with musculoskeletal pain and demonstrated excellent internal consistency (Cronbach α = 0.93), test–retest reliability, and construct validity.^[39]^

### 2.8 Secondary co-morbid symptom outcomes

Our previous study suggests that EA improves pain-associated co-morbidities, such as fatigue and psychological distress^[40]^; thus, we will use validated patient-reported outcomes to measure the effects of acupuncture on fatigue,^[41]^ anxiety/depression,^[42]^ post-traumatic stress disorder (PTSD),^[43]^ and sleep disturbance.^[44]^

The Brief Fatigue Inventory is a validated 9-item instrument that provides a reliable measure of fatigue in cancer populations with Cronbach α of 0.96.^[41]^ Three items ask patients to rate the severity of their worst, usual, or current fatigue during normal waking hours. Six items assess the amount that fatigue has interfered with the daily activities of the patient during the past 24 hours. A composite fatigue severity score can be found by averaging the score obtained on each test item.

The Hospital Anxiety and Depression Scale (HADS) will be used to determine the effects of acupuncture on anxiety and depression. This 14-item, self-administered rating scale has 2 subscales (anxiety and depression), each containing 7 items. Scores of ≥11 on either subscale indicate clinically abnormal psychological morbidity; scores of 8 to 10 indicate borderline cases; and scores of 0 to 7 are considered normal.^[42]^ The reliability, validity, and factor structure of the HADS has been established in cancer patients with Cronbach α of 0.83 (anxiety subscale) and 0.79 (depression subscale).^[45]^

The PTSD Check List–Civilian (PCL-C) is a validated 17-item instrument that corresponds to the Diagnostic and Statistical Manual of Mental Disorders-IV (DSM-IV) criteria for PTSD. It has demonstrated adequate psychometric properties, including internal consistency (Cronbach α = 0.94), test–retest reliability, convergent validity, and discriminant validity.^[46]^ The total score ranges from 17 to 85 with higher scores indicating greater degree of reported stress.

The Pittsburgh Sleep Quality Index (PSQI) is a 19-item instrument that produces a global sleep quality score as well as specific sleep component scores: quality, latency, duration, disturbance, habitual sleep efficiency, use of sleeping medications, and daytime dysfunction. Questions are scored on a 0 to 3 scale over a period of 1 month. The sum of the 7 components yield 1 global score that will be used as a patient-reported outcome for sleep disturbance. Global scores range from 0 to 21 and reflect the number and severity of sleep problems; scores of ≥5 indicate poor sleep quality and high sleep disturbance.^[44]^ The psychometric properties of the PSQI have been established in a variety of populations with Cronbach α ranging from 0.70 to 0.83.^[47]^

The PROMIS-Global Health is a 10-item instrument that has demonstrated reliability and validity as a measure of health-related quality of life.^[36,48]^ It contains 2 domains, mental health (Cronbach α 0.86) and physical health (Cronbach α 0.81).^[48]^

### 2.9 Assessment of outcome expectancy as a predictive variable for acupuncture response

Patient expectancy has been shown to be an important predictor of acupuncture outcomes.^[49]^ The Acupuncture Expectancy Scale (AES) is a 4-item instrument developed by the principal investigator (JJM) to measure outcome expectancy in the context of acupuncture treatment. It has demonstrated reliability and validity in oncology populations with a Cronbach α of 0.95 and is sensitive to change over time in response to acupuncture treatment.^[50]^ It has also been shown to correlate with patient self-efficacy and treatment satisfaction.^[51]^ The score ranges from 4 to 20, with higher scores indicating greater patient expectation of improvement.

### 2.10 Assessment of genetic polymorphisms as predictive variables for acupuncture response

Genetic biomarkers represent an emerging focus of research on pain and responses to treatment.^[52,53]^ In our recent work, we have examined the genetic polymorphisms, Catechol-O-methyltransferase (COMT) and T-cell leukemia 1A (TCL1A).^[54]^*COMT* is an enzyme that regulates dopamine catabolism and other processes associated with the placebo effect, such as reward, pain, memory, and learning.^[55]^ In animal models and clinical studies, acupuncture has been shown to regulate dopamine^[56,57]^ and key brain regions involved in pain, memory, and learning,^[58,59]^ thus providing biological plausibility for the role of COMT in responses to acupuncture. Similarly, *TCL1A* has been shown to be associated with musculoskeletal pain,^[60,61]^ suggesting that it may modulate pain-related processes. Building on this preliminary evidence, we seek to determine whether genetic variants in *COMT* and *TCL1A* predict responses to acupuncture in patients experiencing chronic musculoskeletal pain.

We will collect whole blood samples (4 mL) in an EDTA tube and store them at −80°C in preparation for processing. If a patient does not wish to provide a blood sample, we will offer a saliva collection kit as an alternative, and patients will be asked to collect 2 mL of saliva in Oragene•DISCOVER (OGR-500) collection kits (DNA Genotek Inc.) following the manufacturer's instructions. Saliva specimens will be kept at room temperature until extraction in batches.

Blood and/or saliva specimens will be delivered to MSK's Molecular Epidemiology Laboratory for DNA extraction using the Qiagen QIAamp DNA Blood Kit or the PrepIT-L2P purifier according to the manufacturers’ instructions. Extracted DNA will be quantified and qualified with a Nanodrop ND-8000 to estimate the total DNA and with a Qubit 4.0 (Invitrogen) to estimate the amounts of double stranded DNA. We will conduct genotyping using custom designs and PCR-based genotyping assays (Agena Bioscience, Inc), as we have done in our previous research.^[62–64]^

### 2.11 Covariates

To better characterize our population, we will collect data on sociodemographics (eg, age, education, race/ethnicity), clinical history (eg, tumor stage, cancer treatments, time since diagnoses), and pain medication usage. We will obtain pain medication prescription information by having patients bring in their medication bottles. Additionally, patients will be asked to complete 1 week of daily pain medication diaries at weeks 0, 4, 10, 12, 16, and 24 to calculate weekly average pain medication usage throughout the study time period.

### 2.12 Interventions

The study interventions (EA and BFA) will be delivered by licensed acupuncturists with >5 years of experience in oncology settings. All study acupuncturists will receive a training manual detailing the EA and BFA treatment protocols. The principal investigator (a physician-acupuncturist with extensive oncology experience) will then provide in-person training for each acupuncturist. Before delivering any treatments, all study acupuncturists will be required to be certified by the principal investigator. Acupuncture sessions will be audited bi-weekly to ensure fidelity to study protocols. For quality assurance, acupuncturists will be re-certified twice a year. New acupuncturists who join the study will need to complete the identical training and certification process. These methods to ensure the fidelity of intervention delivery have been successful in our previous acupuncture trial.^[65]^

For the EA intervention, we will use a standardized, semi-fixed protocol developed and tested by our group.^[65]^ The acupuncturist will choose at least 4 local points near the body area with the most severe pain. Additionally, the acupuncturist will choose at least 4 distal points to address the patient's co-morbid symptoms. After sterilizing the skin, the acupuncturist will insert needles (total number between 10 and 20) at appropriate angles and depths, depending on the location on the body and body type of the patient.^[66]^ The acupuncturist will manually manipulate the needles to achieve the “De Qi” sensation. “De Qi” is a local sensation of soreness, numbness, or distension that accompanies the insertion and manipulation of acupuncture needles.^[67]^ The needles at the 4 local points will be electrically stimulated at 2 Hz with a TENS unit. The acupuncturist will leave the needles in place for 30 minutes. After removing the needles, the acupuncturist will apply a sterile cotton-tipped applicator to any areas of bleeding.

For the BFA intervention, the acupuncturist will follow a strictly standardized protocol developed by Niemtzow.^[20]^ Unlike EA, the BFA protocol is not customized to specific pain locations or comorbid conditions. The acupuncturist will clean both ears with alcohol swabs and then place an ASP needle in the Cingulate Gyrus point on 1 ear. Afterwards, the acupuncturist will instruct the patient to walk for a minute, assisting as needed and assessing for any signs of dizziness or lightheadedness indicative of a vasovagal response. After the brief walk, the acupuncturist will evaluate the patient's pain severity. If the reported pain is >1 of 10 and the patient is willing to continue, the acupuncturist will then place another ASP needle in the Cingulate Gyrus point of the other ear. This process is repeated for each of the other ear points: Thalamus, Omega-2, Point Zero, then Shen Men. The acupuncturist will stop placing ASP needles if one of the following conditions is met: pain decreased to 1 or 0 of 10; patient declines further needling due to discomfort; or significant vasovagal response is observed. The total duration of BFA delivery is about 10 to 20 minutes, depending how many ASP needles (up to 10 total) are administered. The needles remain in place for 3 to 4 days. Patients are instructed how to remove the needles safely.

### 2.13 Wait-list control group

Patients in the wait-list control (WLC) group will be followed for a 12-week waiting period; during this waiting period, WLC participants will be contacted by the research coordinator at the same frequency as the acupuncture groups and complete assessments at the same timepoints as the acupuncture groups. WLC patients will continue to receive their standard pain management and medical care as prescribed by their healthcare providers, including analgesic medications. Given the ethical implications of withholding a potentially beneficial treatment, we will offer all WLC patients the option of receiving 10 acupuncture treatments after week 12 (the time of the primary endpoint assessment). The WLC patients will be able to choose whether to receive EA or BFA. The WLC will provide a usual care comparison group so that we can understand the overall magnitude of the acupuncture intervention effects. The WLC will also increase the rigor of our study by controlling for regression to the mean, Hawthorne effect, and the natural history of pain processes in the usual care setting.

### 2.14 Analytic approach

We will perform analysis according to the intention-to-treat (ITT) principle (ie, subjects will be analyzed according to the treatment group to which they were randomly allocated). The change in BPI pain intensity from baseline to Week 12 is the primary endpoint for this study. To maintain the overall type 1 error level at 5% for testing the primary endpoint between study arms, we will use a “gatekeeping” approach to manage the multiple statistical comparisons.^[68]^ We will first separately compare BFA to WLC and EA to WLC using the methods described below. If both acupuncture groups are significantly superior to WLC at significance threshold *P* < .025 (to maintain overall type 1 error at 5% for the 2 tests), we will compare BFA to EA using a noninferiority approach, calculating a 1-sided, 95% confidence interval.

For Aim 1, to compare the effects of acupuncture on patient-reported pain (primary outcome), physical functions, and comorbid symptoms (fatigue, sleep disturbance, anxiety, depression, and PTSD) at week 12, we will use linear mixed models (LMMs)^[69]^ to test differences between treatment arms in score changes from baseline to week 12, with randomization strata as covariates. Specifically, in addition to the randomization strata, the models will adjust for baseline score^[70]^ and will contain assessment time, treatment arm, and the time-by-treatment arm interaction. The interaction term will be used to test whether the treatment arms significantly differ in their changes from baseline. To determine the durability of treatment effects, we will report the change between week 12 and week 24 using ordinary least squares regression in the BFA and EA group separately. For secondary outcomes, we will use similar analytical strategies for physical functions, co-morbid symptoms (fatigue, sleep disturbance, anxiety/depression, PTSD), and quality of life. Additionally, we will use the weekly pain medication diaries to calculate average pain medication usage across the study time for each study participant to determine if acupuncture decreased the use of pain medication.

For Aim 2, to determine the interaction between outcome expectancy and type of needling delivery/stimulation (EA vs BFA) on pain reduction, we will define response to acupuncture therapy as a continuous outcome measured as percent change of the BPI pain intensity score from baseline to week 12. Similar to our approach in our previous study,^[71]^ we will then build a multiple linear regression model with percent reduction in BPI intensity as the dependent variable, and baseline expectancy and treatment group (EA or SA) as independent variables, including the expectancy and treatment group interaction term. The regression coefficient for the interaction term represents the between-group difference of percent reduction in BPI for one unit change in the expectancy score.

For Aim 3, we hypothesize that those participants with either AA in COMT (rs4680) or GG/AG in TCL1A (rs2369049) will be more likely than those without the genetic combination to respond to acupuncture treatments (EA or BFA). To ensure our findings can result in actionable information to guide clinical care, we will calculate percent pain intensity reduction between Week 12 and Baseline for each patient, and we will define a binary variable for clinical response to acupuncture as 30% pain reduction between week 12 and baseline. This binary response variable is consistent with that established by Farrar et al. to be a clinically important change in pain trials.^[31]^ We will perform rigorous quality control for the genotype data and will exclude genotypes that are missing for >15% subjects and subjects with >15% missing SNPs from subsequent analyses. We will tabulate genotype frequencies and perform tests of Hardy-Weinberg Equilibrium (HWE) separately for each race/ethnicity subgroup. To test our primary hypothesis that polymorphisms in COMT and TCL1A are associated with acupuncture response, we will develop multivariable linear regression models with the genotype for each SNP and treatment indictor (EA vs BFA) included as independent variables. A significant association will be claimed if the *P* value for the genotype variable is <0.025 (since we are testing 2 SNPs). In addition to assessing associations with binary pain response, we will also explore associations with changes in the raw scores and percent change. We will code each genotype as the count of minor alleles in all association testing.

### 2.15 Power analysis and sample size

For our Aim 1 sample size/power considerations for comparisons between EA versus WLC and BFA versus WLC, we calculated the smallest standardized effect size (ie, Cohen d) that we will be able to detect with 80% power, given our gatekeeping multiple testing strategy and sample sizes of 144 in each of the acupuncture arms and 72 in WLC. To estimate this smallest detectable effect size, we applied sample size calculations designed for LMMs in the context of RCTs with participant attrition.^[72]^ Using the “power.mmrm” function from the R package “longpower,” we applied the formulas in Lu et al^[72]^ to derive the smallest detectable effect size for the coefficient of the time-by-arm interaction term in our LMM (see Section 2.14), which we transformed to represent the standardized mean difference (ie, Cohen d) between 2 arms in changes in pain intensity from baseline to week 12. Assuming a 20% attrition rate across all arms by week 12, significance threshold of *P* < 0.025, a correlation between baseline and post-treatment assessments of 0.5, and power of 80%, we will be able to detect an effect size of ≥0.48 between either EA versus WLC or BFA versus WLC. This is a moderate effect size which is smaller than that found in our previous pilot trial^[65]^ (effect size of 0.76) and that detected by the meta-analysis conducted by Vickers et al (0.5 between acupuncture and standard care).^[17]^ Thus, our trial is adequately powered to detect such a difference.

As part of our gatekeeping multiple testing procedure, we split our overall type I error rate of 5% evenly between the test of EA versus WLC and the test of BFA versus WLC. If neither, or only one of, EA or BFA is better than WLC at the *P* < .025 threshold, then there is no need for us to evaluate whether BFA is noninferior to EA. On the contrary, if both EA and BFA have significant improvements in pain intensity compared to WLC at the *P* < .025 threshold, then we will proceed to evaluate whether BFA is noninferior to EA. In this gatekeeping scenario, the alphas from both of the comparisons with WLC will propogate to our noninferiority comparison and our overall type I error rate for testing our primary endpoint will be preserved at 5%. Given our sample size, we will have 80% power to find BFA non-inferior to EA with respect to change in BPI pain intensity within a margin of .33 change-score standard deviations (SDs), assuming a 1-sided significance threshold of *P* < .05. We expect this SD to be between 2 and 3, so this margin translates to between 0.67- and 1-point difference in BPI pain intensity reduction. If we are able to demonstrate this, the interpretation would be that BFA is as good as EA with the caveat that we cannot exclude the possibility that EA is slightly better but not to the degree of clinical importance.

Aim 2 hypothesizes that there is a significant interaction between treatment and baseline AES on percent pain intensity reduction. Our preliminary data demonstrate that the regression coefficient for the interaction term between treatment and AES is 8.31 (standard error = 4.21) using percent change in BPI-severity as the dependent variable in a linear regression model including treatment, baseline AES, and their interaction.^[71]^ Using a conservative estimate of correlation (0.2) between the interaction term and the dependent variable, we need a total of 191 subjects to detect this observed interaction effect with 80% power. Thus, our proposed sample size (288 subjects in both EA and BFA groups) is sufficient to detect this observed interaction effect.

For Aim 3, we performed a power calculation to compare response to acupuncture in carriers of AA in rs4680 or GG/AG in rs2369049 versus noncarriers in the EA group. In the preliminary study, 47.2% of 38 patients were carriers; the response rates were 45% in noncarriers and ∼78% in carriers.^[54]^ Therefore, assuming 45% carrier rate, there will be 52 carriers (∼45% of 116 patients) and 64 noncarriers. Assuming a 45% response rate in noncarriers, as shown in the preliminary study, we will have at least 90% power at 5% significance level to detect at least 30% difference in response rates between 52 carriers and 64 noncarriers. Similar power calculation applies to comparison of response to acupuncture in carriers and noncarriers in the BFA group.

## 3 Discussion

Despite advances in medical therapy, chronic pain remains inadequately treated and costs the United States >$600 billion each year in health care expenditures and lost productivity.^[1]^ The opioid crisis has highlighted the devastating public health consequences of chronic pain^[73]^ and sparked growing interest from governmental and medical organizations to integrate nonpharmacologic options into pain management.^[7]^ Our study will provide timely findings in the era of the opioid epidemic to guide the evidence-based delivery of acupuncture to cancer survivors with pain.

Substantial research has established the effectiveness and safety of acupuncture for pain management in general populations^[9,10,17,18]^; however, further research is required to determine its clinical utility for pain conditions in oncology settings.^[15]^ Expected to exceed 20 million by 2026,^[74]^ American cancer survivors represent a rapidly growing, diverse population with high symptom burden that negatively impacts their recovery and quality of life.^[2]^ Our study will help to understand whether two acupuncture techniques, EA or BFA, are effective nonpharmacologic treatment options for pain and common comorbid conditions in this growing population.

Although acupuncture is available at approximately 75% of academic cancer centers in the United States^[75]^ and has demonstrated relatively high levels of acceptability in cancer patients compared to the general population,^[76]^ only an estimated 1.7% to 31% of the oncology population has tried acupuncture,^[77]^ highlighting that important barriers to acupuncture remain. Contrary to EA, the BFA protocol can be learned and delivered by nonacupuncturist clinicians and is notable for its relative ease of administration and scalability. In 2013, the Department of Defense/Veterans Administration Joint Incentive Fund established the Acupuncture Training Across Clinical Settings (ATACS) initiative to develop, pilot, evaluate, and implement a national education program for VA health care providers. By 2016, ATACS had successfully trained >2,700 providers to deliver BFA; of these, approximately 110 were certified to train additional providers in BFA.^[24]^ Despite these successes, the real-world implementation of BFA has been hindered by the lack of high-quality data on the long-term effectiveness and side effects of BFA.^[25]^ Our study will address this evidence gap and provide clarity on the appropriate role of BFA in pain management. If BFA is found to be safe and effective, the implementation efforts by the VA will provide a template to overcome access barriers and increase acupuncture availability across healthcare systems.

The results of our study will need to be considered in the context of several limitations. First, although the study includes diverse cancer types across various stages and is conducted at multiple sites throughout New York and New Jersey, this is a single-center trial at a tertiary cancer center; thus, the results may not be generalizable to other populations. Second, this study does not include a sham acupuncture control group, thus precluding evaluation of the specific efficacy of these interventions; however, previous research has demonstrated that acupuncture produces significantly greater pain reduction than sham controls.^[17,18]^ Third, due to ethical concerns about withholding potentially beneficial treatments from patients with moderate-severe pain, this study allows patients in the WLC to receive acupuncture treatments after the primary endpoint; although this may limit comparison of long-term effects between the acupuncture versus WLC groups, a meta-analysis of 17,922 patients with chronic nonmalignant pain previously showed approximately 90% of the pain-relieving effects of acupuncture were sustained at 12 months relative to controls.^[78]^ Finally, our study design does not allow for crossover between the two acupuncture groups; thus, we will not be able to evaluate whether nonresponders to one acupuncture modality may demonstrate greater improvements with the other modality.

Despite these limitations, this study represents the largest RCT to date to evaluate the comparative effectiveness of two acupuncture techniques (EA and BFA) versus usual care for chronic pain in a diverse cancer population, presenting a unique opportunity to advance nonpharmacological pain management in the era of the opioid epidemic. Our investigation of clinical predictors of acupuncture response (ie, patient expectations, genetic biomarkers) will also help to inform more personalized approaches to treatment. Other strengths of our study include large sample size, rigorous design, 6-month follow-up, and inclusion of diverse cancer types across multiple stages of disease. This research has the potential to shift pain management from a one-size-fits-all paradigm towards a personalized model that integrates individuals’ expectations and genetic biomarkers to deliver “precision” acupuncture with maximal benefit to cancer survivors suffering from chronic pain.

## Acknowledgments

The authors thank the patients, oncologists, nurses, and clinical staff at all study sites for their contributions to this study.

## Author contributions

Kevin T. Liou: Investigation, writing – original draft, writing – review and editing. Ray Baser: Formal analysis, writing – review and editing. Sally A.D. Romero: Project administration, writing – review and editing. Jamie Green: Project administration, data curation, writing – review and editing. Q. Susan Li: Project administration, data curation, writing – review and editing. Irene Orlow: Resources, methodology, writing – review and editing. Katherine S. Panageas: Methodology, formal analysis, writing – review and editing. Jun Mao: Conceptualization, funding acquisition, investigation, supervision, writing – review and editing.
